# Using virtual reality to define the mechanisms linking symptoms with cognitive deficits in attention deficit hyperactivity disorder

**DOI:** 10.1038/s41598-019-56936-4

**Published:** 2020-01-17

**Authors:** Aman Mangalmurti, William D. Kistler, Barrington Quarrie, Wendy Sharp, Susan Persky, Philip Shaw

**Affiliations:** 1 Neurobehavioral Clinical Research Section, Social and Behavioral Research Branch, NHGRI/NIH, Bethesda, United States; 2 Immersive Virtual Environment Testing Unit, Social and Behavioral Research Branch, NHGRI/NIH, Bethesda, United States

**Keywords:** Psychology, Translational research

## Abstract

The mechanisms underpinning attentional deficits are only partially understood. Here we ask if shifts in a child’s field of view (FOV) act as a mediator between symptoms of attention deficit hyperactivity disorder (ADHD) and associated cognitive anomalies, particularly in attentional processes. Real time measurement of shifts in FOV were obtained on 85 children (mean age 9.4 (SD 1.9) years; 45 with DSM 5-defined ADHD) as they completed the continuous performance task in a “virtual classroom”. We extracted measures reflecting focused and selective attention across the task, along with diffusion modelling of latent cognitive processes of information uptake, response conservativeness and non-decision time. Mediation analyses showed that shifts in FOV partially mediated the relationship between hyperactive impulsive symptoms and both poor focused attention and information uptake. Performance accuracy decreased and shifts in FOV increased during the task, but these changes over time did not differ by symptom severity. Employing virtual reality and mediation analysis, we implicate shifts in FOV as a mechanism linking symptoms of ADHD and deficits in focused attention and in the gathering of information to make decisions. The identification of mediating mechanisms might provide new targets for intervention.

## Introduction

Why do children with attention deficit hyperactivity disorder (ADHD) fail to pay attention? How can we link clinical symptoms of ADHD, reported by parents or children, to objective, quantifiable cognitive deficits? Here, we aim to identify the mechanisms that link ADHD symptoms with deficits in performance during a widely used probe of attention—the continuous performance task (CPT). A large literature delineates how objective deficits in this demanding task are associated with the symptoms of ADHD^1–4^ and that these deficits are partly rectified by psychostimulant medication^5–7^.

One possible mechanism to link ADHD symptoms with deficits in attention-demanding tasks is rapid shifts in a child’s field of view (FOV) resulting from eye or head movements. Concentrated attention on a target usually corresponds with the object falling at or near the center of the FOV^8–10^. Thus, shifting of the FOV away from targets could readily disrupt performance on attention-laden tasks. There is some empirical evidence supporting this concept. First, studies that track eye movements demonstrate that children with ADHD fail to maintain visual focus on a target, making excessive, off-target eye movements, or saccades^11–14^. However, maintaining gaze on a target without any other demands lacks the complexity of most tasks that require focused attention, and this paradigm does not probe selective attention. A second research strand monitors head movement in an effort to track shifts in a child’s FOV^15,16^. For example, one study found that compared to typically developing children, those with ADHD show excessive and distinct patterns of head movement while performing attention-demanding tasks^17^. However, the possibility of links between such shifts in FOV due to head movement and specific deficits in attentional skills is unexplored. The importance of identifying mediating mechanisms, such as shifts in FOV, lies partly in the possibility that they may be amenable to remediation.

Several models have been proposed to account for the links between ADHD symptoms, particularly hyperactivity-impulsivity, and objective, quantifiable attentional deficits. Some emphasize anomalies in the arousal of the central nervous system. Early versions posited CNS hypoarousal in ADHD which leads not only to deficient sustained attention but also to compensatory increases in motor activity to boost arousal^18^. More recent models argue for a generalized dysregulation of arousal in ADHD, most readily detected during tasks that required long periods of sustained focus^19,20^. Hyperactivity is seen as an attempt at autoregulation, as the child tries to attain stable levels of arousal. Building on this work, Sergeant argued that anomalies in arousal (defined as phasic alertness) are accompanied by deficits found in other ‘energetic’ pools: energy used to meet task demands (effort) and tonic changes in processes impacting response speed (activation)^21^. Sonuga-Barke and colleagues refined this model to argue physiological arousal per se is less pathophysiologically pertinent than the management of arousal into task-related activity^22^. Aligned with these arousal models, several theories tie deficient focused attention to anomalies in the coordinated, ‘intrinsic’ patterns of neural activity that emerge spontaneously when a subject is not engaged in task oriented behavior^23,24^. Here, ADHD is conceptualized as an imbalance between these intrinsic networks, particularly the default mode network, prominent during internally directed thought, and the networks supporting cognitive control and attention^25,26^. By this reasoning, lapses in attention are due to a loss in the counterbalanced activity of the default mode and task positive networks.

By contrast, cognitive models have emphasized the roles of both impaired response inhibition and a specific vulnerability to the presence of singleton distractors during attention-demanding tasks. Impairement in response inhibition has been characterized as a core feature of ADHD^27,28^. Though response inhibition has been conceptualized to include three subcomponents including action withholding and action cancellation, it is interference control, the process of protecting self-directed responses from competing events, which is linked by some evidence to the observed deficits of attention in ADHD. Some studies, using the Simon and Flanker tasks, suggest that deficits in interference control lead to an initial misallocation of attentional resources and a subsequent inability to garner task-specific information^29,30^. A second body of research explores the effect of unique, salient, but otherwise extraneous environmental features, known as a singleton distractors, in capturing attention during attention-demanding tasks^31,32^. While such singleton distractors provide an intuitively plausible explanation for deficient attention, there is mixed evidence for a specific effect in individuals with ADHD^33–35^. These mechanisms thus provide at best a partial explanation of deficient attention. Here, we advance the field by examining how another mechanism—shifts in a child’s field of view—might also explain how ADHD symptoms are linked to deficits in observed behavior and underlying cognitive processes, using a virtual reality paradigm.

Virtual reality is used for three reasons. First, it immerses the child entirely in a realistic setting that is completely controlled, and all participants experience the same visual and auditory reality. Importantly, the nature of distracting events is experimentally determined, unlike ‘real world’ settings where unexpected interruptions and distractions occur. Secondly, the head-set that is used to ‘present’ the virtual reality can simultaneously be used to monitor shifts in a child’s FOV. This allows moment by moment shifts in FOV to be tied to behavioral performance. Finally, virtual reality can readily incorporate well-established probes of attention, such as the continuous performance task^3,36,37^. We used a virtual reality tool developed by Rizzo and colleagues in which the child is asked to complete the CPT presented on a virtual chalkboard surrounded by a virtual teacher and virtual classmates^38^. The use of a virtual reality paradigm allows an examination of dynamic measures of changing behavior during the task, particularly how the magnitude of shifts in FOV might increase over the duration of a long, demanding task. Measuring performance over time is important as it has been argued that a core feature of ADHD is a more rapid decline in performance particularly during effortful, attention demanding tasks (i.e. there is a time by diagnosis interaction effect)^39^. As noted above, some theoretical models have linked this time-locked decline in performance to deficits in ‘energetic’ pools, particularly the allocation of effort over time^21,40^.

Most analyses of the CPT focus on directly observed measures, often summarized by factors that explain most variance in performance. Such analyses have returned orthogonal domains of attention, specifically focused attention (characterized by accurate, consistently fast responses) and selective attention (characterized mainly by the accurate discrimination of targets and non-targets)^41,42^. Others have used the CPT to infer latent, underlying cognitive processes that elucidate how response time and accuracy vary between subjects. One such approach is diffusion modeling, and we employ a version, the EZ diffusion model. This model is suited to the version of the CPT we used, which has a modest number of trials and sometimes a low error rate. The EZ diffusion model infers three latent cognitive processes from response times, response time variance and accuracy rates. The first process is ‘information uptake’, which refers to the rate at which the individual acquires the information necessary to make a forced choice. The second is ‘response conservativeness’, denoting the degree of certainty an individual has to attain before making a choice. The final is the ‘non-decision time’, which indexes how long it takes to accomplish all of the cognitive tasks not directly involved in the discriminative choice. Here, we examine the associations between ADHD symptoms and both directly observed measures of attention and inferred underlying cognitive processes, and further relate these measures to shifts in FOV.

**Figure 1:**
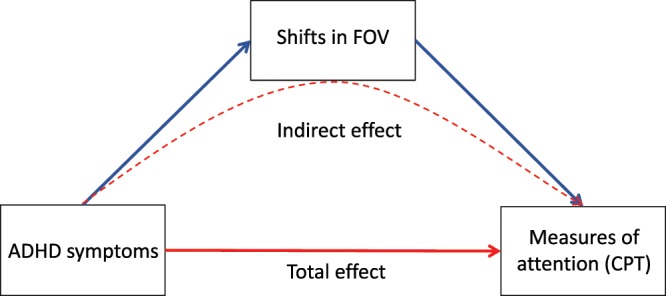
Diagram of the main hypothesis. We expect that shifts in field of view (FOV) will mediate, or help explain, the relationship between symptoms of the disorder and measures of attention derived from the continuous performance test.

We use a virtual reality paradigm to test the hypothesis that shifts in a child’s FOV will partly explain the association between ADHD symptoms and observed deficits in attentional performance on the CPT – see Fig. 1. We had no prediction on whether symptoms of hyperactivity/impulsivity or symptoms of inattention would be more strongly associated with impaired performance on the CPT. Given the limited literature, we also did not hypothesize on which inferred latent cognitive processes would be most impaired in ADHD.

## Methods

### Participants

Eighty-five children participated. The general inclusion criteria were (1) age between 6 and 12 years and (2) an Intelligence Quotient of 75 or greater, defined using age-appropriate versions of the Wechsler Abbreviated Intelligence Scales^43,44^. General exclusion criteria were (1) neurological disorders known to affect movement, (2) the presence of gross anatomic anomalies on MRI, and (3) psychiatric disorders other than ADHD, oppositional defiant disorder or conduct disorder. In all children, we ascertained the symptoms of inattention (minimum of zero and maximum of nine) and hyperactivity/impulsivity (minimum of zero and maximum of nine) using the Diagnostic Interview for Children and Adolescents, a clinician administered interview with parents^45^. Medication histories were obtained from parents. Forty-five children met criteria for ADHD, of whom 13 were being treated in the community with psychostimulant medications. Working memory was estimated from the digit and spatial-span tasks of the Wechsler Intelligence Scale for Children^46^; and processing speed from the visual matching and decision speed subtests in the Woodcock Johnson Battery^47^. The procedures were approved by the Institutional Review Board of the National Human Genome Research Institute, and this study was conducted in accordance with the approved guidelines. All parents gave informed, written consent and children gave written and verbal assent.

### Virtual classroom

A virtual classroom was presented to children via the “ClinicalVR: Classroom CPT” program and a head mounted, immersive virtual reality device (Sony HMZ-T1). This task was developed by Rizzo and colleagues^38^ and adapted by Digital MediaWorks (http://www.dmw.ca/). The CPT is presented on a chalkboard in the front of the virtual room. The objective is to detect and respond to the target stimulus “K”, if and only if it follows the letter “A” (the AK-CPT). Responses are made by pressing the left mouse button. Main errors consist of responses made to non-target letters (e.g. “X”) or to the target “K” when it is not preceded by the letter “A” but another letter (e.g. “B” then “K” or “C” then “K”). A total of 500 stimuli are presented in 5 blocks with an interstimulus interval of 1300–1400 milliseconds, 100 of which are the target “K” after the presentation of “A”. As the CPT proceeds, a series of classroom-based distractors are systematically presented, such as whispering classmates, paper airplanes being thrown and virtual school staff entering the room. To the extent a child turns his/her head, either to view these distractors or for other reasons, the CPT-relevant stimuli on the chalkboard move out of the center of the FOV.

Shifts in the child’s FOV were captured using the Intersense Inertiacube 3, an inertial measurement unit (IMU) that possesses a 3 degree of freedom (3-DOF) gyroscope and a sampling rate of 180 Hz to record head rotations within 0.25 degrees of accuracy. These measurements are communicated to the virtual program where they are utilized to rotate the in-game camera, providing the perceptual effect of adjusting gaze or rotating one’s head within the virtual environment. Movement was measured in the horizontal (‘yaw’) and vertical planes (‘pitch’) and tilt (‘roll’). These measurements are centered to a zero-reference line from the participant’s position within the virtual classroom to the center of the active virtual chalkboard. The total extent of movement away from the central fixation point in each axis was determined for each of the five blocks, and also summed across the entire task. The task was well tolerated with no child reporting nausea due to the simulated environment. Data, from consenting participants, can be accessed upon request of the authors.

### Analysis

For the entire task, we considered behavioral measures that reflected reaction times, their variability and the type and frequency of errors—see Supplemental Table 1. Skew in the distribution of scores was reduced using a log transformation when appropriate. As the behavioral measures were correlated (most r > 0.3), we reduced the dimensionality of these data by extracting principle components with varimax rotation, retaining factors with eigenvalues greater than one. Diffusion modeling used EZ diffusion tools to extract parameters reflecting latent cognitive processes from the mean and variance of correct reaction times and accuracy, namely information uptake, response conservativeness and non-decision time^48^.

Mediation analyses were used to determine if shifts in the FOV mediated the relationship between symptoms of hyperactivity-impulsivity and inattention and our measures of attention from the CPT. We include a dimensional approach, considering symptom number in our analysis, rather than solely diagnostic categories. Treating symptoms as a quantitative trait boosts power as traits contain more information about between-individual trait variability than a dichotomous diagnostic category. This approach is also in line with epidemiological evidence showing that symptoms of ADHD are distributed throughout the population^49,50^. Examining the two symptom dimensions separately is also in keeping with reports that components of the CPT show different associations with symptoms of hyperactivity-impulsivity and symptoms of inattention^3,51^. Mediation analyses determine the degree of attenuation in the association between ADHD symptoms and the CPT performance variables in a model that includes the proposed mediator (shifts in the FOV). If this association is attenuated significantly, a significant indirect effect exists of symptoms on attentional performance through mediating shifts in FOV^52^. We used a bootstrapping approach, with 5000 resamples, to test the significance of the mediator. This provides a bias corrected confidence interval for estimates, and significant mediation is indicated when the 95% confidence interval does not cross zero^53^. We tested for the effects of sex, age and psychostimulant medication using moderated mediation models. We allowed these factors to interact with all paths in the mediation model and removed each interaction if it did not show significant moderation of that pathway. For each model, the indirect effect of symptoms on attention measures through the mediator is expressed as a linear function of the moderator (sex, age, and medication) in the model. The slope of the line relating the indirect effect to the moderator is termed the index of moderated mediation. If the bootstrap confidence intervals of this index does not cross zero, then the moderation effect is taken to be significant. We used the PROCESS Macros for SPSS developed by Preacher and Hayes (processmacro.org/).

Changes in performance and the degree of shift in FOV over the five blocks were examined using repeated measures ANOVA. Block by block performance was the within-subject factor and diagnosis was the between subjects factor. The performance measures for each block were: accuracy, reaction time, reaction time variability and the extent of yaw, pitch and roll. We did not consider variables such as impulsive errors to targets only or reaction time variability to targets only, as these were too infrequent within each block to be considered in block by block analysis.

## Results

### Indices of behavioral performance and correlations with symptoms

Demographic and clinical characteristics of the 85 children are given in Table 1.

**Table 1:** Demographic and clinical characteristics.

	Typically developing	ADHD	Test of group difference
Male; Female	26; 14	36; 9	Fisher’s exact test p = 0.15
Age: mean(SD) in years	9.6 (2.0)	9.1 (1.8)	t(85) = 1.3, p = 0.2
IQ: mean [SD]	112 (13)	110 (16)	t(80) = 0.46, p = 0.65
Hyperactive-impulsive symptoms: mean (SD)	1.1 (1.8)	5.0 (3.0)	t(85) = 7.5, p < 0.0001
Inattentive symptoms: mean (SD)	1.5 (2.0)	6.2 (2.0)	t(85) = 10.8, p < 0.0001
Psychostimulant medication	N/A	13 (29%)	

First, a principal components analysis on the behavioral data across the entire task extracted three factors—see Supplemental Table 1. The first factor (explaining 33% of variance) pertained to poor focused attention, with loadings from measures that indicated accurate, consistently fast responses. A second factor of ‘impulsive responding’ reflected responses to non-targets, and a third factor of ‘inattentive responding’ reflected the target occurring without the correct preceding stimulus. The second and third factors pertain to selective attention and explained 21% and 16% of the variance respectively. Parameters reflecting latent cognitive processing were calculated using diffusion modeling (information gathering, response conservativeness, and non-decision time). Correlations between the observed and inferred measures are shown in Supplemental Table 2.

### Correlations between symptoms, shifts in FOV, and cognition

ADHD symptoms were significantly correlated with poor focused attention (for hyperactivity-impulsivity r = 0.48, p < 0.0001; for inattention r = 0.31, p = 0.004) but not with selective attention. Hyperactive-impulsive symptoms were also associated with the latent cognitive process of information gathering (r = 0.41, p = 0.0001) and at a nominal level of significance to response conservativeness (r = 0.26, p = 0.01). Inattention correlated only with information gathering (r = −0.23, p = 0.03)—Supplemental Table 2.

**Figure 2:**
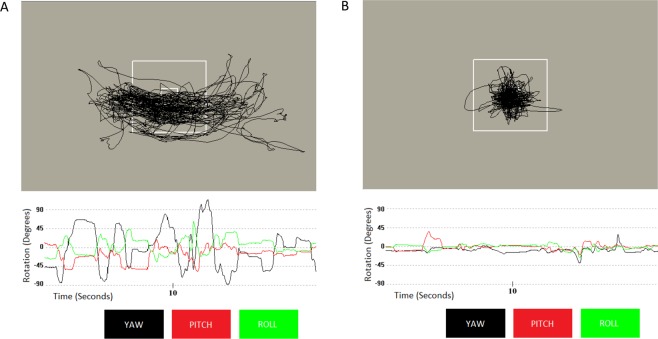
(**A**) This panel shows the shifts in the field of view, tied to head movement, in a child with nine symptoms of hyperactivity/impulsivity. The outer white box indicates ± 45° and the inner box ± 2.5°. The lower panel shows the degree of movement in each plane (yaw, itch and roll) over a 20 second period. (**B**) Shifts in field of view over the same period in a different child who had no symptoms of hyperactivity/impulsivity.

We found that the total amount of shifts in FOV (measured by yaw, pitch, roll) was associated with hyperactivity-impulsivity (r = 0.29, p = 0.008), but not inattention (r = 0.18, p = 0.1). To illustrate this association, we show the shifts in FOV during the task for two children; one child with nine symptoms of hyperactivity-impulsivity (the maximum number) and the other free from symptoms—see Fig. 2.

### Do shifts in FOV mediate the relationship between symptoms and cognitive anomalies?

**Figure 3:**
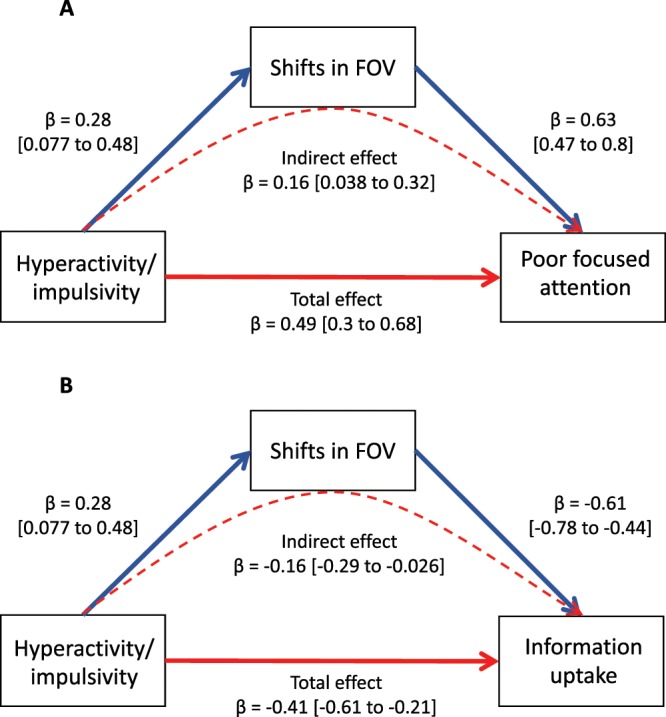
(**A**) Mediation analyses, demonstrating how shifts in field of view (FOV) partly mediate the relationship between hyperactive-impulsive symptoms and poor focused attention as well as (**B**) information uptake. The strength of the ‘total’ unmediated path between symptoms and attention and the indirect effect of symptoms and shifts in FOV on attention are given, and the standardized beta coefficients with 95% confidence intervals for each path are shown.

Mediation analyses determined whether the associations between ADHD symptoms and observed performance and latent cognitive processes (information gathering, boundary separation, and non-decision time) were mediated by shifts in FOV–see Supplemental Table 3. Symptoms of hyperactivity-impulsivity remained a significant predictor of poor focused attention after controlling for shifts in FOV (standardized $\beta$ = 0.33, CI 0.17 to 0.49, p = 0.0001) but the strength of this relationship was attenuated compared to the unmediated model (standardized $\beta$ = 0.49, CI, 0.3 to 0.68, p =  < 0.0001). This is consistent with shifts in FOV partially, but not completely, mediating the relationship between symptoms and poor focused attention. In keeping with these results, the indirect effect of symptoms on poor focused attention through the mediator was significant (standardized $\beta$ = 0.16, CI, 0.038 to 0.32). Overall, this mediated model accounted for 52% of the variance in poor focused attention (F[2, 82] = 44, p < 0.0001)—see Fig. 3A.

Turning to inattentive symptoms, their relationship with poor focused attention, mediated through FOV (standardized $\beta$ = 0.2, CI, 0.033 to 0.36), was only marginally lower than the unmediated relationship (standardized $\beta$ = 0.31, CI, 0.1 to 0.52). The indirect effect was not significant (standardized $\beta$ = 0.11, CI, −0.019 to 0.24). Thus, the relationship between inattentive symptoms and poor focused attention was not mediated by shifts in FOV. The shifts in FOV also did not emerge as a significant mediator between either symptom dimension and the two factors that reflected ‘selective’ attention (represented by impulsive and inattentive responding).

Shifts in FOV also emerged as a significant mediator of the association between hyperactivity-impulsivity and information uptake (indirect effect: $\beta$ = −0.16, CI, −0.29 to −0.026). Overall, this mediated model accounted for 45% of the variance in poor focused attention (F[2, 82] = 33, p < 0.0001) —see Fig. 3B. Shifts in FOV did not mediate the relationship between inattention and information gathering, response conservativeness or non-decision time.

The mediation patterns were similar for both males and females (the index of moderated mediation was not significant for models that included sex as a moderator). Similarly, the mediation pathways did not differ significantly between children who were and were not taking psychostimulant medication—see Supplemental Table 4.

We asked if other neuropsychological features might act as mediators of the associations between hyperactive-impulsive symptoms and poor focused attention. Significant mediation emerged for processing speed ($\beta$ = −0.053, CI −0.15 to −0.0017), though not for general intelligence (indirect effect $\beta$ = 0.0057, 95% CI −0.037 to 0.060) or working memory ($\beta$ = 0.017, CI −0.012 to 0.085). Significant mediation of the association between symptoms of hypeactivity-impulsivity and information uptake was also observed for processing speed ($\beta$ = 0.073, 95% CI 0.015 to 0.18), but not general intelligence ($\beta$ = −0.006, 95% CI −0.067 to 0.043) or working memory ($\beta$ = −0.032, 95% CI −0.12 to 0.028). We did not find significant mediation of the relationship between symptoms and selective attention or any other latent cognitive process.

### Symptoms and change during the task in behavior and FOV

**Figure 4:**
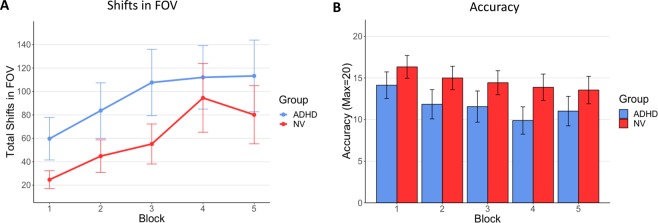
(**A**) The total amount of shift in FOV across each block by diagnosis. (**B**) The block by block change in accuracy for the ADHD and typical groups. (95% confidence intervals are shown.).

The amount of shifts in FOV (summing pitch, yaw and roll) increased block by block in a linear manner (F[4,336] = 20.88, p < 0.000001), and the shift in FOV overall was significantly greater in the ADHD group (F[1,83] = 5.69, p = 0.019)—See Fig. 4A. However, the steady increase in shifts in FOV during the task was not associated with diagnosis (F[4,332] = 0.48, p = 0.75), nor with symptoms of inattention (F[4,332] = 0.52, p = 0.72) or hyperactivity-impulsivity (F[4,332] = 0.76, p = 0.55). Thus, while those with ADHD had more shifts in FOV overall, the increasing FOV seen during the task did not vary by diagnosis: both groups showed a steady increase over time.

A similar finding emerged for other behavioral measures. Thus accuracy decreased across the five blocks in a linear fashion (F[3,336] = 18.74, p < 0.000001) and the ADHD group had lower overall accuracy (F[1,83] = 7.89, p = 0.006)—Fig. 4B. However, the decline in accuracy over time was not greater in the ADHD group (F[4, 332] = 1.27, p = 0.28). Reaction time but not reaction time variability showed an increase across the task (linear trend for RT, F[4,336] = 2.72, p = 0.03 linear trend for RT variability, F[4,336] = 1.91, p = 0.11), but neither showed an overall diagnostic difference (for both RT and RT variability, group difference p > 0.1) and there was no block by diagnosis interaction (for RT, F[4,332] = 1.11, p = 0.35, for RT variability F[4,332] = 0.74, p = 0.57). In short, we found neither the diagnosis of ADHD nor its symptoms were associated with accelerated decline in performance or increase in shifts in FOV over the duration of the task. Given the lack of significant associations between symptoms and change over the blocks in accuracy and shifts in FOV, we did not proceed to mediation analyses.

## Discussion

Using virtual reality, we show that shifts in a child’s FOV in part explain the link between hyperactive-impulsive symptoms and deficits in focused attention and information gathering. This link was specific as shifts in FOV did not contribute to deficits pertaining to selective attention nor to other latent cognitive processes, such as a conservative response style. While it is not the focus of the current study we also find that processing speed partially explains the same links between hyperactive-impulsive symptoms and both poor focused attention and information gathering.

We illustrate how a portable and non-invasive technology can probe the mechanisms linking ADHD symptoms to its associated cognitive profile. In interpreting these findings, we consider neurocognitive models that have been proposed to account for links between symptoms of hyperactivity-impulsivity and deficits in focused attention as well as the reduction in the rate of information uptake observed in children with ADHD. The first models, mentioned earlier, emphasize how deficits in CNS arousal—either pervasive under-arousal or dysregulated arousal—can lead directly to deficits during tasks demanding sustained focus, and to hyperactive, sensation seeking before, as an attempt to boost or regulate anomalous arousal^18,19^. Aligned with these models are models that view lapses in attention as arising from a loss in the counterbalanced activity of the brain’s default mode and attention/cognitive control networks^25,26^. Such dysregulated inter-network connectivity is thought to cause subjects to repeatedly disengage from task-oriented processing. In turn such disengagement is thought to lead to longer and more variable response times. We find that more variable, slower response times loaded onto a factor reflecting poor focused attention, and this factor was correlated with ADHD symptom severity. It is possible that the increased shifts of FOV we detect in ADHD might also stem from dysregulated interactions between the default mode and task positive networks. Such atypical interactions could cause subjects to disengage in a task and then re-engage their attentional focus elsewhere^54^.

Others have directly linked anomalies in motor (head) control with attentional deficits, arguing that the head movements that drive shifts in FOV are a facet of postural instability in ADHD^16^. In turn this postural instability may reflect cerebellar-cortical dysfunction. In support of this model, Teicher and colleagues showed that children with ADHD exhibited three times as much head movement as typical children during a continuous performance task, and there were diagnostic differences in the patterns of head movement^17^. However, this study by Teicher and colleagues did not directly examine how the degree of head movement mapped onto specific attentional deficits. We did define these links and show that shifts in FOV due to head movement were tied to deficits in focused but not selective attention. The next step is to define the neural substrates of this finding. Neuroimaging techniques such as Near Infrared Spectroscopy that allow relatively free head movements may be particularly useful in this context^55^.

Changes in FOV also mediated the relationship between symptoms of hyperactivity-impulsivity and the rate of information uptake necessary for decision making. Intuitively, shifts in FOV could disrupt the gathering of information, with the underlying deficit perhaps related to postural instability, as argued earlier^17^. Similarly, changes in FOV that are caused by dysregulated inter-network connectivity, which in turn are tied to lapses in focus on relevant stimuli, could also result in atypical information gathering.

The current analysis did not show a relationship between shifts in FOV and selective attention, indicating that there are other mechanisms at play. Failures in selective attention, in which a child fails to attend and respond to targets rather than non-targets can be construed as failures in inhibitory control. In turn, deficits in inhibitory control have been linked with the failure of lateral prefrontal cortical centers to provide control over behaviors, such as eye movements, generated at lower striatal and collicular levels^56,57^. Future studies are needed to determine whether impairments in selective attention might be underpinned by anomalies in this lateral-prefrontal-striatal-collicular circuitry.

Our analysis also pointed to processing speed as a partial mediator for the associations between symptoms of hyperactivity/impulsivity and poor focused attention as well as information uptake. Deficits in several measures of processing speed have been observed in children with a primarily inattentive, but not combined, ADHD subtype^58^. Perhaps unexpectedly, we find an association between more symptoms of hyperactivity-impulsivity and greater processing speed. We speculate that mediation by processing speed may represent quick decision making that precludes necessary information uptake for correct decision making. Further investigation is warranted to better understand the link between symptoms of hyperactivity-impulsivity, processing speed, and functional and clinical outcomes.

It has been speculated that individuals with ADHD have deficits in several ‘energetic’ pools: phasic alertness (arousal), energy used to meet task demands (effort) and tonic changes in processes impacting response speed (activation)^21^. Deficits in effort are thought to be reflected in the rapid decline in performance seen over long, demanding tasks such as the CPT^3^. However, we did not find that ADHD was tied to a more rapid decline in performance, nor to a more rapid increase in shifts in FOV. Rather the deficits relative to unaffected controls were maintained at an essentially constant level throughout the task. This pattern—a difference in intercepts, but not slopes—argues that ADHD is not characterized by a more rapidly exhausted capacity for effortful self-control; rather there is a ‘fixed’ deficit which is immediately apparent and not exacerbated by time on task^59^. However, it is also possible that our virtual classroom, with its many extraneous distractors, served to activate the arousal system. The use of a version of the CPT without any such distractors might be better suited to detect time-on-task effects, particularly those tied to ADHD.

This study has several limitations. First, shifts in FOV might ideally be monitored by combining the measurement of head position with eye-tracking. However, this is technically challenging to implement and head movements appear a more reliable index of how shifts in attention are tied to action^60–62^. Secondly, some participants with ADHD were treated with psychostimulant medication, which can affect performance on the CPT acutely^6,63^. To mitigate the possibility of this medication confounding results, it was stopped a day prior to testing. Additionally, our moderation analyses found that usual treatment with psychostimulants did not impact the overall pattern of results. Thirdly, the age range included in the study spans significant developmental variation in cognition. We did not, however, find moderating effects of age on the mediation results—see Supplemental Table 4. Finally, we only used one attentional task, and our findings would be bolstered by demonstrating a similar lack of association between hyperactive-impulsive symptoms and other probes of selective attention, such as the Erisken flanker task^64^.

By identifying a mechanism that mediates the link between ADHD symptoms and deficient focused attention, we provide a possible target for remediation. For example, it follows from the postural instability model that interventions to improve the control of head movement might boost a child’s skill in sustaining attention. Other interventions might include redirecting the child’s FOV to pertinent stimuli, perhaps through continuous feedback on the direction of gaze or head position. In conclusion, we illustrate how virtual reality can probe the mechanisms linking the symptoms of ADHD with its characteristic cognitive profile. Identification of these mediating mechanisms could provide new targets for intervention.

## Supplementary information

Supplemental Info.
