# Supplementary material for: Using virtual reality to define the mechanisms linking symptoms with cognitive deficits in attention deficit hyperactivity disorder
Source: Sci Rep. 2020 Jan 17;10:529. doi: 10.1038/s41598-019-56936-4 (PMC6969149; doi:10.1038/s41598-019-56936-4)
Supplement: Supplementary file 1 — Supplemental Info. [file 41598_2019_56936_MOESM1_ESM.docx]

**Supplemental information for:**

**Using virtual reality to define the mechanisms linking symptoms with cognitive deficits in attention deficit hyperactivity disorder.**

Aman Mangalmurti^1^*, William D Kistler^2^*, Barrington Quarrie^1^, Wendy Sharp^1^, Susan Persky^2^ and Philip Shaw^1^§

* Both authors contributed equally to this work.

Supplemental Table 1: The loadings of the 12 behavioral measures of the CPT onto the three principal components is given.

Key: RT-reaction time; “all other errors of commission” refers to errors of commission not included in the values of “Impulsive error: response to warning letter before target appears”, “Impulsive error: to perceptually similar non-target (i.e. X after A)”, “Inattentive error: log of responses to target in absence of a preceding stimulus (K without A)”, or “Inattentive error: respond to K following a H”.

|  | Component | | |
| --- | --- | --- | --- |
|  | Poor focused attention | Selective attention | |
|  |  | 2: Impulsive responding | 3: Inattentive responding |
| Correct hits | **-.846** | .104 | .246 |
| RT variability for commission errors | **.749** | .113 | .302 |
| Log of all other errors of commission | **.738** | .200 | .327 |
| RT variability to targets | **.736** | -.278 | .236 |
| Variability of correct hits across blocks A-E | **.695** | .229 | -.359 |
| Impulsive error: response to warning letter before target appears | **.598** | .331 | .455 |
| RT commission errors | **.500** | -.227 | .426 |
| Impulsive error: to perceptually similar non-target (i.e. X after A) | -.087 | **.846** | .249 |
| Impulsive error: to letter following the warning letter other than K or X | .170 | **.843** | .128 |
| RT to targets | .006 | **-.810** | .021 |
| Inattentive error: log of responses to target in absence of a preceding stimulus (K without A) | .220 | .073 | **.818** |
| Inattentive error: respond to K following a H (log) | -.058 | .294 | **.749** |

One type of impulsive error (Impulsive error: response to warning letter before target appears) and the RT for commission errors cross-load onto two PCs. We repeated the PCA dropping the cross-loading items. As shown below in Supplemental Table 1b, the new factors still reflected poor focused and selective attention with similar item loadings.

Supplemental Table 1b: The principal components that emerge when cross-loading items are dropped.

|  | Component | | |
| --- | --- | --- | --- |
|  | Poor focused attention | Selective attention | |
|  |  | 2: Impulsive responding | 3: Inattentive responding |
| Correct hits | **-0.861** |  |  |
| Log of all other errors of commission | **0.745** |  | 0.384 |
| RT variability to targets | **0.742** |  |  |
| RT variability for commission errors | **0.739** |  | 0.306 |
| Variability of correct hits across blocks | **0.705** |  | -0.336 |
| Impulsive error: to perceptually similar non-target (i.e. X after A) |  | **0.856** |  |
| Impulsive error: to letter following the warning letter other than K or X |  | **0.848** |  |
| RT to targets |  | **-0.816** |  |
| Inattentive error: log of responses to target in absence of a preceding stimulus (K without A) |  |  | **0.847** |
| Inattentive error: respond to K following a H (log) |  |  | **0.774** |

Running mediation analyses using these PCs resulted in very similar results. For example, in the analysis reported in the main manuscript, shifts in FOV emerged as a significant mediator of the relationship between hyperactivity-impulsivity and poor focused attention (but not between inattention and poor focused attention). These findings held (see Supp Table 1c below) when using the PCs derived as above.

Supplemental Table 1c: The mediation results using PCs derived from the full range of measures available in the CPT, and when using only items that did not cross load. Similar mediation findings emerged.

|  | Original poor focused attention component | Poor focused attention (with no cross-loading items) |
| --- | --- | --- |
| Indirect mediating effect of FOV (on hyperactivity-impulsivity to poor focused attention) | standardized $\beta$=0.16  [0.038 to 0.32] | standardized $\beta$=0.16  [0.028 to 0.31] |
| Indirect mediating effect of FOV (on inattention to poor focused attention) | standardized $\beta$=0.11  [-0.019 to 0.24] | standardized $\beta$=0.11  [-0.013 to 0.27] |

Supplemental Table 2: Pearson correlations between symptoms dimensions, shifts in field of view (FOV), factors reflecting poor focused attention, selective attention, and diffusion decision model variables

|  | Hyperactivity | Inattention | Total movement | Poor focused attention | Impulsive responding | Inattentive responding | Non-decision time | Information uptake | Boundary separation |
| --- | --- | --- | --- | --- | --- | --- | --- | --- | --- |
| Hyperactivity |  |  |  |  |  |  |  |  |  |
| Inattention | .54(<.0001)* |  |  |  |  |  |  |  |  |
| Total movement | .29(0.008)* | .18(.099) |  |  |  |  |  |  |  |
| Poor focused attention | .49(<.0001)* | .31(.004)* | .65(<.0001)* |  |  |  |  |  |  |
| Impulsive responding | -.13(.23) | .089(.42) | .036(.74) | <.0001(1) |  |  |  |  |  |
| Inattentive responding | -.057(.6) | .13(.22) | -.058(.6) | <.0001(1) | <.0001(1) |  |  |  |  |
| Non-decision time | -.11(.23) | -.14(.2) | -.31(.003)* | -.43(<.0001)* | -.55(<.0001)* | -.14(.21) |  |  |  |
| Information uptake | -.41(.0001)* | -.23(.031) | -.62(<.0001)* | -.86(<.0001)* | .072(.52) | .18(.095) | .23(.037) |  |  |
| Boundary separation | .26(.015)* | .06(.58) | .023(.83) | .24(.03) | -.44(<.0001)* | .24(.029) | -.29(.0069)* | -.064(.56) |  |

|  |  | **Total effect** | | **Indirect effect** | | **Direct effect** | |
| --- | --- | --- | --- | --- | --- | --- | --- |
|  |  | β | CI | β | CI | β | CI |
| **Hyperactivity/ Impulsivity** | Poor focused Attention | 0.49 | 0.3 to 0.68* | 0.16 | 0.038 to 0.32* | 0.33 | 0.17 to 0.49* |
|  | Selective Attention 1: Impulsive responding | -0.13 | -0.35 to 0.09 | -0.15 | -0.38 to 0.07 | 0.021 | -0.023 to 0.10 |
|  | Selective Attention 2: Inattentive responding | -0.057 | -0.28 to 0.16 | -0.013 | -0.11 to 0.05 | -0.044 | -0.27 to 0.018 |
|  | Information Uptake | -0.41 | -0.61 to -0.21* | -0.16 | -0.29 to -0.026* | -0.25 | -0.42 to -0.076* |
|  | Response Conservativeness | 0.26 | 0.053 to 0.47* | -0.017 | -0.11 to 0.047 | 0.28 | 0.059 to 0.5* |
|  | Non-decision time | -0.11 | -0.33 to 0.10 | -0.088 | -0.22 to -0.022* | -0.027 | -0.24 to 0.19 |
| **Inattention** | Poor focused Attention | 0.31 | 0.1 to 0.52* | 0.11 | -0.019 to 0.24 | 0.20 | 0.033 to 0.36* |
|  | Selective Attention 1: Impulsive responding | 0.088 | -0.13 to 0.31 | 0.0037 | -0.032 to 0.052 | 0.085 | -0.14 to 0.31 |
|  | Selective Attention 2: Inattentive responding | 0.13 | -0.083 to 0.35 | -0.015 | -0.087 to 0.018 | 0.15 | -0.072 to 0.37 |
|  | Information Uptake | -0.23 | -0.45 to -0.021* | -0.11 | -0.23 to 0.018 | -0.12 | -0.3 to 0.047 |
|  | Response Conservativeness | 0.06 | -0.16 to 0.28 | 0.0023 | -0.042 to 0.07 | 0.058 | -0.17 to 0.28 |
|  | Non-decision time | -0.14 | -0.36 to 0.077 | -0.054 | -0.15 to 0.0025 | -0.085 | -0.3 to 0.13 |

Supplemental Table 3: Total, indirect, and direct effects for the mediating effect of shifts in field of view on the association between symptoms of ADHD and measures of attention.  Standardized beta coefficients with 95% confidence intervals (CI) are given. *Effects are significant if the bootstrapped confidence intervals do not cross zero.

|  | Sustained attention | | | | | | Information uptake | | | | | |
| --- | --- | --- | --- | --- | --- | --- | --- | --- | --- | --- | --- | --- |
|  | Gender | | Age | | Psychostimulant Medication | | Gender | | Age | | Psychostimulant Medication | |
|  | Index of Moderated Mediation | CI | Index of Moderated Mediation | CI | Index of Moderated Mediation | CI | Index of Moderated Mediation | CI | Index of Moderated Mediation | CI | Index of Moderated Mediation | CI |
| Interaction: path a only | -0.22 | -0.49 to -0.0075 | -0.028 | -0.26 to 0.29 | -0.36 | -0.92 to 0.059 | 0.22 | 0.0023 to 0.48* | 0.028 | -0.25 to 0.29 | 0.36 | -0.053 to 0.9 |
| Interaction: paths a, b | -0.24 | -0.54 to 0.033 | 0.02 | -0.23 to 0.34 | -0.36 | -1.1 to 0.29 | 0.25 | -0.052 to 0.54 | -0.0012 | -0.27 to 0.27 | 0.35 | -0.064 to 0.97 |
| Interaction all paths: | -0.24 | -0.55 to 0.033 | 0.017 | -0.24 to 0.35 | -0.36 | -1.12 to 0.3 | 0.26 | -0.051 to 0.56 | 0.0005 | -0.27 to 0.27 | 0.33 | -0.10 to 0.86 |

Supplemental Table 4: We tested for the effects of gender, age, and psychostimulant medication using moderated mediation models. We allowed age, gender and medication status to interact with all paths in the mediation model, and removed each interaction if it did not show significant moderation of that pathway. For each model, the indirect effect of symptoms on attention measures through the mediator is expressed as a linear function of the moderator (age, gender and medication) in the model. The slope of the line relating the indirect effect to the moderator is termed the index of moderated mediation. If the bootstrap confidence intervals of this index does not cross zero, then the moderation effect is taken to be significant. Gender status moderated the relationship of the “a” path only, in the mediation of the association between symptoms of hyperactivity and information uptake by shifts in FOV
